# Genetic Markers in *S.* Paratyphi C Reveal Primary Adaptation to Pigs

**DOI:** 10.3390/microorganisms8050657

**Published:** 2020-04-30

**Authors:** Satheesh Nair, Maria Fookes, Craig Corton, Nicholas R. Thomson, John Wain, Gemma C. Langridge

**Affiliations:** 1 Gastrointestinal Bacteria Reference Unit, Public Health England, Colindale, London NW9 5EQ, UK; satheesh.nair@phe.gov.uk; 2 Wellcome Trust Sanger Institute, Hinxton, Cambridge CB10 1SA, UK; mcf@sanger.ac.uk (M.F.); chc@sanger.ac.uk (C.C.); nrt@sanger.ac.uk (N.R.T.); 3 Norwich Medical School, University of East Anglia, Norwich NR4 7UQ, UK; 4 Microbes in the Food Chain, Quadram Institute, Norwich Research Park, Norwich NR4 7UQ, UK; Gemma.Langridge@quadram.ac.uk

**Keywords:** host adaptation, convergent evolution, genome degradation, genomic lesions

## Abstract

*Salmonella enterica* with the identical antigenic formula 6,7:c:1,5 can be differentiated biochemically and by disease syndrome. One grouping, *Salmonella* Paratyphi C, is currently considered a typhoidal serovar, responsible for enteric fever in humans. The human-restricted typhoidal serovars (*S.* Typhi and Paratyphi A, B and C) typically display high levels of genome degradation and are cited as an example of convergent evolution for host adaptation in humans. However, *S.* Paratyphi C presents a different clinical picture to *S.* Typhi/Paratyphi A, in a patient group with predisposition, raising the possibility that its natural history is different, and that infection is invasive salmonellosis rather than enteric fever. Using whole genome sequencing and metabolic pathway analysis, we compared the genomes of 17 *S.* Paratyphi C strains to other members of the 6,7:c:1,5 group and to two typhoidal serovars: *S.* Typhi and Paratyphi A. The genome degradation observed in *S.* Paratyphi C was much lower than *S.* Typhi/Paratyphi A, but similar to the other 6,7:c:1,5 strains. Genomic and metabolic comparisons revealed little to no overlap between *S.* Paratyphi C and the other typhoidal serovars, arguing against convergent evolution and instead providing evidence of a primary adaptation to pigs in accordance with the 6,7:c:1.5 strains.

## 1. Introduction

Standardisation in the classification and fine typing of *Salmonella* in the mid-20th century led to a revolution in our understanding of the biological properties, particularly host associations, of this heterogenous group of animal pathogens [1,2]. For *Salmonella enterica* there are six defined subspecies. Nearly all isolates from humans are from subspecies I, in which more than 1500 serotypes can be distinguished by their cell wall (O) and flagella (H) antigens [3]. The combination of O:H1:H2 is known as the antigenic formula and each unique combination is given a name. For example (using the simplified antigenic formulae used by front line diagnostic laboratories) 9:d:- (where “-“ means no antigen) identifies *S.* Typhi isolates and 2:a:1,5 is *S.* Paratyphi A. Both of these serovars are human-restricted and cause enteric fever. The situation, however, is more complex for *S.* Paratyphi B where its antigenic formula, 4:b:1,2 is shared by several *Salmonella* capable of causing mild to severe disease [4]. The 4:b:1,2 group can be split into subtypes using metabolic ability (e.g., tartrate utilisation), but these groups do not map to specific host species. For example, the human restricted *S.* Paratyphi B *sensu stricto* is always tartrate negative, but other 4:b:1,2 tartrate negative strains are highly diverse and are collectively known as *S.* Java. To date, only sequence typing has been able to define the phylogroup of tartrate negative strains that are human restricted [4]. Currently all *Salmonella* with the antigenic formula 4:b:1,2 are categorised as dangerous pathogens [5], but this may change as evidence linking the sub-types to clinical phenotypes accumulates. 

The fourth *Salmonella* serotype considered to be a human restricted enteric fever pathogen is *S.* Paratyphi C; this belongs to a highly variable group with the antigenic formula 6,7:c:1,5, for which subtyping has never been fully resolved [6,7]. The most commonly isolated member of this group, *S.* Choleraesuis, has been divided into biotypes (Table 1): *sensu stricto* and Kunzendorf have a limited ability to grow using single sugars as a carbon source and differ only in their ability to produce H_2_S from sulphates—a test which is difficult to standardise for routine diagnostic use. 

*S.* Choleraesuis var. Decatur, on the other hand, has a greater metabolic capacity but is also far more genetically diverse [8]. Current biotyping schemes often lead to errors in identification [8], and so molecular tests have been developed using multi locus sequence typing (MLST) [6]. However, the mapping of these sequence types to phylogenetically related clusters is not clean and the association with animal hosts even less so [8]. The utility of these biotypes is therefore disputed. 

The host restricted biotypes within the 6,7:c:1,5 group, (*S.* Paratyphi C to humans and *S.* Typhisuis to pigs) are clearly distinguishable from *S.* Choleraesuis; this has led to the hypothesis that the adaptation of salmonellae to humans by *S.* Typhi, Paratyphi A, B and C, is the result of convergent evolution of a diverse set of *Salmonella* adapting to survive and transmit between human hosts [7]. Comparative genomic studies on *S.* Typhi and Paratyphi A support this theory [9,10], but for *S.* Paratyphi B and C the evolutionary trajectory is less clear. Sequencing of ancient DNA from humans suggests that the 6,7:c:1,5 group of *Salmonella* were far more common in the past than now and were part of a historic clade (Ancient Eurasian Super Branch) which falls within the much larger extant *S. enterica* diversity. The early members of this clade cluster with their pig-adapted modern counterparts [11].

Clinically, the disease caused by *S.* Typhi and Paratyphi A is indistinguishable [12], but the evidence from a search of the accessible published literature that *S.* Paratyphi C causes only enteric fever is less clear. Rather, the clinical descriptions range from opportunistic infection resembling sepsis [13,14,15,16] to classic enteric fever [17]. This presentation, in some cases, is very similar to the infection caused in humans by the pig-adapted *S.* Choleraesuis [18]. The clinical picture is confused by difficulties in identifying *S.* Paratyphi C in the laboratory, but it is possible that this human restricted pathogen has undergone very different selective pressures to *S.* Typhi and Paratyphi A. If *S.* Paratyphi C has evolved convergently with *S.* Typhi and Paratyphi A, then the genome sequence should reveal this through the accumulation, across serotypes, of genetic changes that cause lesions in the pathways associated with host adaptation [19]. Here, we investigate this by describing the genomic lesions present in the genomes of 58 isolates from the 6,7:c:1,5 group, and comparing the evolutionary dynamics of this important group with those reported for *S.* Typhi and Paratyphi A.

## 2. Materials and Methods 

### 2.1. Isolates

All 6,7:c1,5 strains used in the study are listed in Table 1, along with accession numbers for the raw sequence data. We assembled a collection of 6,7:c1,5 isolates for genome sequencing and supplemented these with publicly available sequences where necessary. 

### 2.2. DNA Preparation

All isolates were cultured on non-selective agar (LB agar, Difco, Oxford, United Kingdom) for purity checking, and in non-selective broth (LB broth, Difco) for DNA extraction. DNA was extracted using a genomic DNA extraction (Sigma, Gillingham, United Kingdom) and sequenced using the Illumina Genome Analyzer II (Cambridge, United Kingdom), as previously described [20].

### 2.3. Genomes from Databases

A search was performed in Enterobase [21] for entries with the antigenic formula 6,7:c:1,5. An additional 2 genome sequences were identified and included in this study, *S.* Choleraesuis var. Decatur SARB70 (ERR3482081) and *S.* Paratyphi C RKS4594 (CP000857). 21 *S.* Typhi genomes, CT18 (accession AL513382), Ty2 (AE014613) and 19 from [22], and 2 *S.* Paratyphi A genomes, ATCC 9150 (CP000026) and AKU_12601 (FM200053) were also used.

### 2.4. Reference Genome Assembly

For the *S.* Typhisuis 61-6 and *S.* Paratyphi C 66-8 reference genomes, a high-quality sequence was assembled using data from two sequencing platforms. DNA was sequenced on both 454 Roche GS FLX Titanium (paired end library with 3kb insert; Connecticut, United States) and the Illumina Genome Analyzer II (200–300bp standard paired end library run in one lane for 37 cycles). Illumina sequences were assembled using Velvet [23] and combined with 454 sequences using Newbler (Roche). The combined assemblies were converted to Gap4 databases [24], to guide gap closure based upon 454 read pair information. ABACAS [25] was used to order and orient the fragmented assemblies against the complete genomes of *S.* Enteritidis P125109 (accession AM933172) and *S.* Choleraesuis SC-B67 (AE017220), enabling many small repeat regions to be correctly assembled. Finally, iCORN [26] was used to correct the assembled sequences using the Illumina data and checked in the Gap4 database. Initial genome annotation was carried out using annotation transfer via RATT [27] from *S.* Choleraesuis SC-B67. Genome assemblies are available under the project accession PRJEB37271.

### 2.5. Sequence QC

Where necessary, sequence data were trimmed using Trimmomatic (Galaxy v0.38.0) [28] with sliding window trimming of 4;20, leading and trailing trimming at quality 3 and appropriate adapter clipping (GAII or HiSeq).

### 2.6. Comparative Genomics

The identification of genomic lesions (mutations resulting in premature stop codons, frameshifts or insertion/deletions relative to an intact version of the gene), and comparison between genomes, was carried out using Artemis Comparison Tool (v10) [29]. For each identified lesion, the appropriate reference genome annotation was amended to accurately reflect the mutation, and therefore allow inclusion of these genomic features in downstream analysis. Velvet assemblies were generated for all isolates sequenced in this study. Per cluster, all mutations leading to genomic lesions in the reference genome were checked for their presence in at least 2 other draft genome assemblies from that cluster. The comparators (*S.* Choleraesuis, RKS1235 and RKS1249; *S.* Paratyphi C, 6610 and 664; *S.* Typhisuis, 38K and 871997) were chosen for their divergent positions within the phylogeny. Genomic lesions were deemed core if the same mutation was present in both, or variable if the mutation was absent in one or both sequences. OrthoMCL [30] with default parameters was used to determine orthologues between the three 6,7:c:1,5 genomes, *S.* Typhi CT18 and *S.* Paratyphi A AKU_12601. *S.* Enteritidis P125109 was also used as a comparator. All core disrupted genes were compared via orthology to identify which were shared or unique to each cluster. Core genomic lesions were also identified for *S.* Typhi across 19 genomes and *S.* Paratyphi A across the 2 publicly available genomes at the time.

### 2.7. Pathway Comparison

Lists of disrupted genes were overlaid onto the metabolic pathway overview of the *S.* Typhi CT18 pathway/genome database [31] to identify disrupted pathways and transport reactions. Disrupted pathways were compared between the 5 *Salmonella* to produce an UpSet plot [32].

## 3. Results and Discussion

### 3.1. Reductive Evolution and Genome Degradation

**Figure 1:**
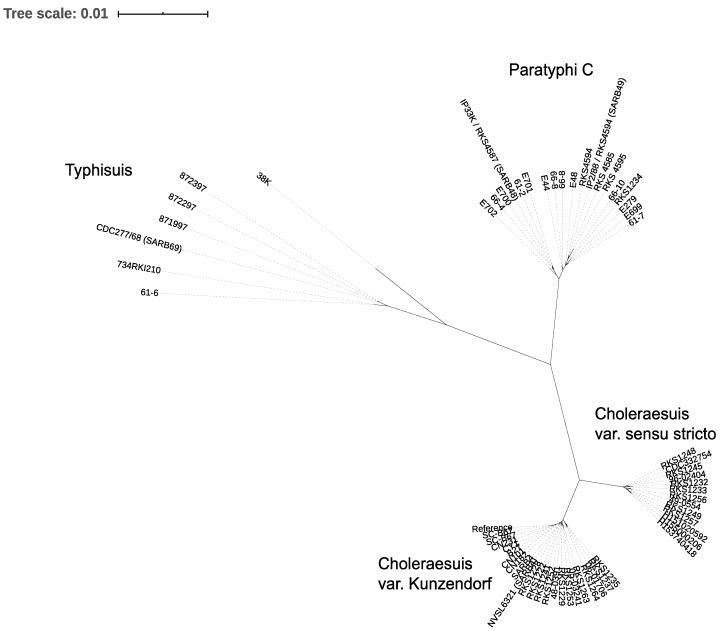
Phylogenetic clusters of 6,7:c:1,5 *Salmonella.* Unrooted core SNP phylogeny using *S.* Choleraesuis SC-B67 as a reference.

We generated a core SNP phylogeny from the genomes of 58 isolates to investigate the relationship between related 6,7:c:1,5 biotypes of *S. enterica* subspecies I (Figure 1). These included 17 isolates of *S.* Paratyphi C which spanned a range of 57 years and were collected from Europe, Africa and the Middle East (Table S1). 

The two *S.* Decatur genomes showed more SNP variation between them than was found across all of the remaining biotypes (Figure S1), and so they were removed from the phylogeny. It is generally accepted that organisms classified as *S.* Decatur represent a very diverse group which should be considered separate from other 6,7:c:1,5 *Salmonella*. The remaining strains grouped into four phyloclusters (Figure 1, supported by hierBAPS analysis, Table S1). We also prepared an extended phylogeny, including an additional 116 assemblies from Enterobase, which confirmed the phylogenetic relationships were robust (Figure S1). The separation of these clusters was matched to the currently accepted classification scheme: 1, *S.* Typhisuis; 2, *S.* Paratyphi C; 3, *S.* Choleraesuis var. *sensu stricto;* 4, *S.* Choleraesuis var. Kunzendorf. This phylogeny confirmed that the isolates included in this study were typical of the 6,7:c:1,5 group, and also allowed us to select representative genomes for comparative analyses. For this analysis, the two biotypes of *S.* Choleraesuis were considered together because the core variation across the two groups was similar to that seen across the *S.* Typhisuis group, and this study focused on the human adaptation of *S.* Paratyphi C, *S.* Choleraesuis var. *sensu stricto* and var. Kunzendorf are both associated with pigs. 

In bacteria restricted to a niche, such as infection of the human host, the accumulation of mutations that disrupt gene sequences (termed “pseudogenes”) is well described [10]. The term pseudogene typically infers a loss of gene function but that is not always accurate, as many of these changes (deletions, insertions or major amino acid substitutions) can cause a change of function [33,34], and so here we use the term genomic lesion (GL) to infer changes that are predicted to have an impact on phenotype but do not necessarily cause loss of function. The association of genomic lesions with host adaptation is an active research area [35]; however, grouping genomic lesions by functional impact remains challenging. We have therefore treated all GLs (mutations resulting in premature stop codons, frameshifts or insertion/deletions relative to an intact version of the gene) as equal. We found that the number of GLs per serotype was much greater for *S.* Paratyphi A and *S.* Typhi than for *S.* Paratyphi C, suggesting a potentially shorter evolutionary time period for accumulation. However, the presence of *S.* Paratyphi C DNA found in ancient human samples [36,37], coupled with the descriptions of *S.* Paratyphi C as being very common in more ancient periods of human history suggests that the effective population size was at least as large as *S.* Typhi. This, in turn, suggests a different selective pressure driving the preservation of metabolic diversity, by the removal of genomic lesions from the population. For *S.* Paratyphi C then, a history of colonisation and transmission between varied environments seems more likely than one of being restricted to the human host. However, it is possible that *S.* Paratyphi C is at an early point along the pathway to human restriction, and so we looked for signs of early adaptation to the human host.

**Figure 2:**
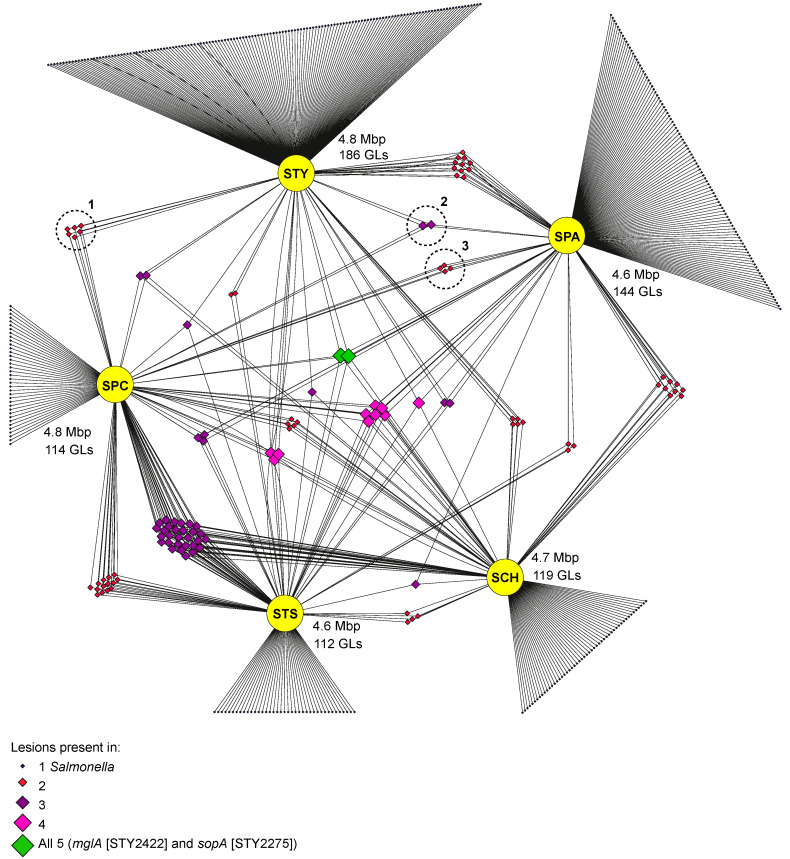
Distribution of disrupted genes. Network of shared and unique disrupted genes in *S.* Paratyphi C (SPC), *S.* Typhisuis (STS), *S.* Choleraesuis (SCH), *S.* Paratyphi A (SPA) and *S.* Typhi (STY). Yellow circles are false nodes representing each *Salmonella*. Diamonds indicate a single genomic lesion; size and colour indicate how many *Salmonella* share that lesion. Black lines connect *Salmonella* to genomic lesions. Lesions unique to each *Salmonella* are shown as fans around the false nodes. Mbp, megabase pairs; GL, genomic lesion. Dashed circles indicate lesions shared between SPC and STY and/or SPC and SPA. 1: *torC*, *ratC*, STY4541, STY2432, STY1834, STY1781; 2: *slrP* and *fliB*; 3: STY4472, STY4044, STY1408, STY1353.

We performed comparative genomics within and between multiple genome sequences of *S.* Paratyphi C, *S*. Typhisuis and *S.* Choleraesuis, to determine if genome degradation was evident. At the time of sequencing, a high-quality contiguous reference genome only existed for *S.* Choleraesuis SCB67; we therefore generated references for both *S.* Paratyphi C and *S.* Typhisuis (Table S1). Within each biotype, a core set of genomic lesions was identified by comparison of the reference with two other members of that group, selected to represent the diversity within the group (Table S2). Any lesion that was absent from one or more of the sequences was considered variable and not included. All the 6,7:c:1,5 biotypes sequenced here, showed similar levels of genome degradation, with just over 100 genomic lesions seen in each genome representative (Figure 2). When compared with the core genomic lesions of the human restricted serotypes, it was evident that the scale of genome degradation was much greater in the two classic human restricted serotypes *S.* Typhi (186 lesions) and Paratyphi A (144 lesions). To understand the impact of these differences in genome degradation, we investigated the possible functional consequences of these genomic lesions.

### 3.2. Genomic Lesions are Functionally Distinct

**Figure 3:**
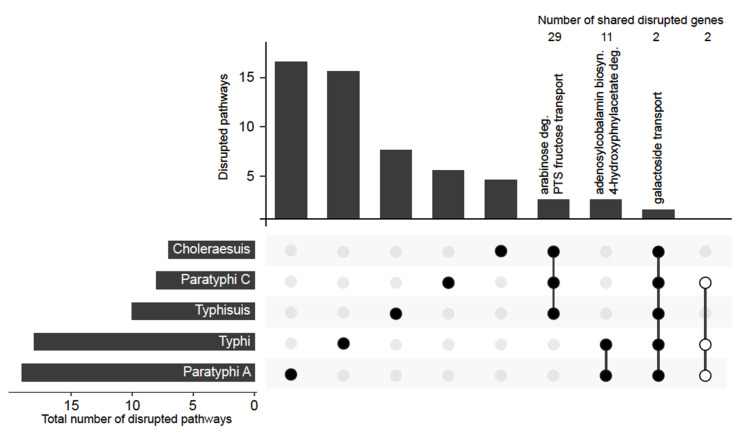
Distribution of disrupted pathways. Annotated UpSet plot of shared and unique disrupted pathways. Number of genes disrupted in pathways given above the columns (includes multiple genes disrupted in same pathway). Open circles: no disrupted pathways are shared between *S.* Typhi, *S.* Paratyphi A and *S.* Paratyphi C.

Genomic lesions were located in metabolic pathways according to a pathway/genome database generated for *S.* Typhi (Table S3). This enabled us to determine which (potentially different) lesions affected the same pathways or transporters between the three 6,7:c1,5 representatives and *S.* Typhi/Paratyphi A (Figure 3). The intersection of affected pathways and transporters revealed that *S.* Paratyphi C had no lesions in pathways/transporters that were also degraded in *S.* Typhi or Paratyphi A (Figure S2). However, there were two genomic lesions that were common to all three 6,7:c1,5 representatives and *S.* Typhi/Paratyphi A: *sopA* and *mglA*. The former encodes a Type Three secretion system (T3SS) effector protein secreted by the T3SS encoded on *Salmonella* Pathogenicity Island 1 (SPI-1), and the latter encodes a galactoside transporter. SPI-1 effectors are associated with the invasion of host intestinal cells and subsequent enteritis [38], and so the disruption of SopA through genomic lesion in all of the *Salmonella* investigated here is not surprising. Disruption of *mglA* has previously been reported in other host-adapted *Salmonella* [39], which combined with our findings suggests that the gene is linked to the former generalist lifestyle of all of these adapted salmonellae. 

For the 6,7:c:1,5 biotypes we saw 29 genomic lesions, two of which impacted two shared pathways/transporters: arabinose degradation and a PTS fructose transport system, which have not been linked to bacterial colonisation of pigs previously. This most likely reflects their shared ancestry but may be the result of selection for colonisation of a shared habitat. We therefore examined the exact nature of the mutations causing these genomic lesions: the same base pair change at the same site was considered to be inheritance whereas a different mutation in the same gene (or pathway) was considered to show selection indicative of convergent evolution. The lesions affecting arabinose degradation and PTS fructose transport were identical and thus considered inheritance. This leaves open the possibility that the ancestor of *S.* Paratyphi C evolved as a pig pathogen, which then crossed the species barrier into humans. If this was the case, it seems likely that *S.* Paratyphi C has crossed from pigs to become an opportunistic human pathogen that has subsequently become restricted to the human host. *S.* Paratyphi C and *S.* Choleraesuis cause a similar pathology in humans but *S.* Paratyphi C has lost the ability to infect pigs. This hypothesis is supported by the reporting of *Salmonella* genomes in ancient DNA studies [11]. The genomes of *Salmonella* from 400 to 900 years ago cluster with modern *S.* Paratyphi C, but the older genomes (1600–5000 years old) cluster more closely with the pig-adapted *S.* Choleraesuis. This supports the hypothesis that the ancestor of *S.* Paratyphi C was adapted to pigs and that host restriction in humans actually represents an evolutionary dead end, explaining why *S.* Paratyphi C is so rare today.

Five pathways/transporters were found to be disrupted in both *S.* Typhi and Paratyphi A, of which two were shared by them alone. For *S.* Typhi/Paratyphi A, approximately 25% of their genome is shared through a recombination event, and the mutations causing GLs in this region of the genome accordingly have identical base pair changes [9]. However, outside of this shared region there are many mutations casing GLs that are clearly the result of convergent evolution: independent mutations linked to the disruption of the same metabolic pathways. Our analysis agrees with this finding, we identified genomic lesions in 11 genes and two pathways present only in *S.* Typhi and Paratyphi A, once all shared lesions with the 6,7:c:1,5 biotypes had been considered. Of these lesions, some were identical mutations and some were independent [9], suggesting convergent evolution by both selection for independent mutations and horizontally acquired characteristics. 

### 3.3. Anaerobic Respiration Intact in S. Paratyphi C

The link between host adaptation (leading towards restriction) in *Salmonella* is perhaps best described for the metabolic functions associated with tetrathionate. Tetrathionate reduction allows the bacterial cell to carry out anaerobic respiration, this in turn increases the growth rate in the anaerobic environment of the animal gut and generates specific metabolic end products which stimulate inflammatory diarrhoea and in turn transmission between hosts [40]. The loss of anaerobic metabolism through the disruption of tetrathionate reduction is a hallmark of host adaptation, where a balance between pathogenicity and long-term colonisation of the host is the evolutionary strategy, rather than population expansion through rapid transmission. In Salmonella, disruption of anaerobic respiration is seen in *S.* Typhi, *S.* Paratyphi A and the *S.* Gallinarum/Pullorum group (restricted to birds) [10,19]. For *S.* Paratyphi C no such disruption was seen, the anaerobic respiration pathways were intact. In fact, only two genomic lesions were shared solely between *S.* Paratyphi C, *S.* Typhi and *S.* Paratyphi A (*slrP* and *fliB*), and these did not map to metabolic functions. 

## 4. Conclusions

For *S.* Typhi and Paratyphi A, there is a clear genomic signal that suggests convergent evolution during adaptation and eventual restriction to the human host. In this study of 17 *S.* Paratyphi C genomes, we found: (i) the level of genome degradation caused by mutational disruption was much lower than in other host-restricted *Salmonella*, (ii) the metabolic pathways involved did not match the pathways disrupted in the human restricted *Salmonella*, and (iii) the acquisition of mutations appeared to be through inheritance from the ancestor with *S.* Choleraesuis, rather than by the selection of randomly occurring errors of DNA replication. In short, this suggests that convergent evolution does not explain the host restriction of *S.* Paratyphi C. Indeed, since anaerobic respiration is intact in *S.* Paratyphi C, we hypothesise that pathogenicity would be more similar to non-typhoidal *Salmonella* than to the enteric fever group. This has implications for both biological understanding and risk assessment for safety in clinical laboratories.

## Figures and Tables

**Table 1:** Differentiation by biochemistry of 6,7:c:1,5 *Salmonella.*

	Dulcitol	H2S	Mucate
Paratyphi C	+	+	−
Choleraesuis var. sensu stricto	−	−	−
Choleraesuis var. Kunzendorf	−	+	−
Choleraesuis var. Decatur	+	+	+
Typhisuis *	−	−	−

Adapted from the Kauffman White scheme [3]. * Typhisuis is *d*-tartrate negative in contrast to the other four.

## Supplementary Materials

Supplementary materials can be found at https://www.mdpi.com/2076-2607/8/5/657/s1; **Figure S1.** Extended phylogeny. Strains in red indicate those sequenced as part of this study. Strains in black are from Enterobase assemblies with > 60x coverage from Illumina sequencing. All *S.* Typhisuis and *S.* Paratyphi C genome assemblies from Enterobase with >60x coverage (representing 10 and 41 strains, respectively). We then selected a diverse geographical and temporal range of assemblies from Choleraesuis Kunzendorf (25), sensu stricto (15) and those defined only as Choleraesuis (25), all with > 60x coverage. 14 strains appear to be distantly related, possibly indicating misclassification. All other strains conform to the same phylogenetic relationship demonstrated by the strains sequenced in this study. **Figure S2.** Network of disrupted pathways and transporters. Yellow circles are false nodes representing each *Salmonella*. Diamonds indicate a single disrupted pathway (connected by straight lines) or transporters (connected by wavy lines); size and colour indicate how many *Salmonella* share that disrupted pathway/transporter. Disrupted pathways/transporters unique to each *Salmonella* are shown as fans around the false nodes. Table S1. 6,7:c1,5 strains in this paper. Table S2. Genomic lesions affecting genes: *S.* Paratyphi C (SPC), *S.* Typhisuis (STS), *S.* Choleraesuis (SCH), *S.* Paratyphi A (SPA) and *S.* Typhi (STY). Table S3. Genomic lesions affecting pathways: *S.* Paratyphi C (SPC), *S.* Typhisuis (STS), *S.* Choleraesuis (SCH), *S.* Paratyphi A (SPA) and *S.* Typhi (STY).
