# Supplementary material for: Genetic Markers in S. Paratyphi C Reveal Primary Adaptation to Pigs
Source: Microorganisms. 2020 Apr 30;8(5):657. doi: 10.3390/microorganisms8050657 (PMC7285187; doi:10.3390/microorganisms8050657)
Supplement: Supplementary file 1 [file microorganisms-08-00657-s001.zip › Figure S1 extended phylogeny.pdf]

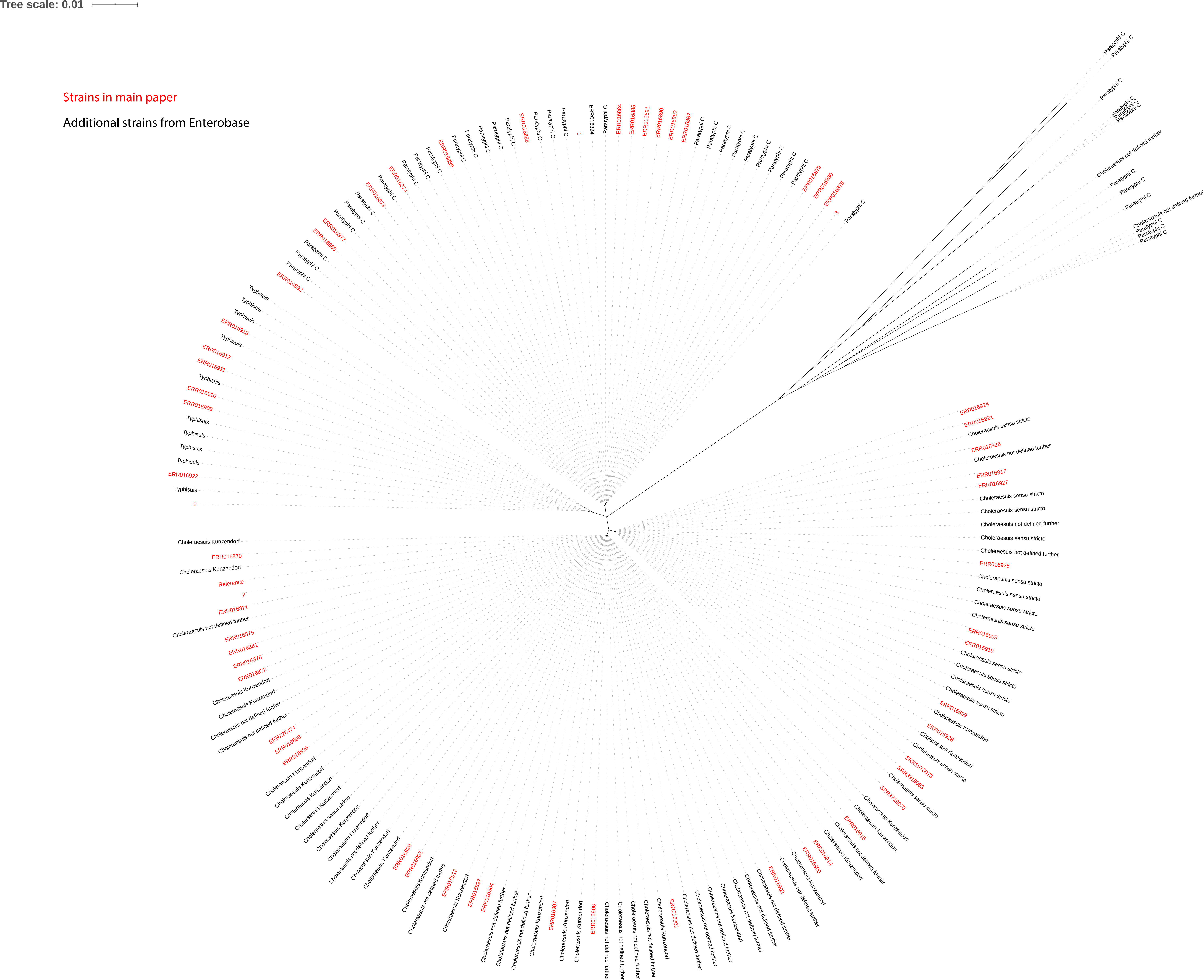
Tree scale: 0.01
Strains in main paper
Additional strains from Enterobase
