# Supplementary figures and images for: Genetic Markers in S. Paratyphi C Reveal Primary Adaptation to Pigs
Source: Microorganisms. 2020 Apr 30;8(5):657. doi: 10.3390/microorganisms8050657 (PMC7285187; doi:10.3390/microorganisms8050657)

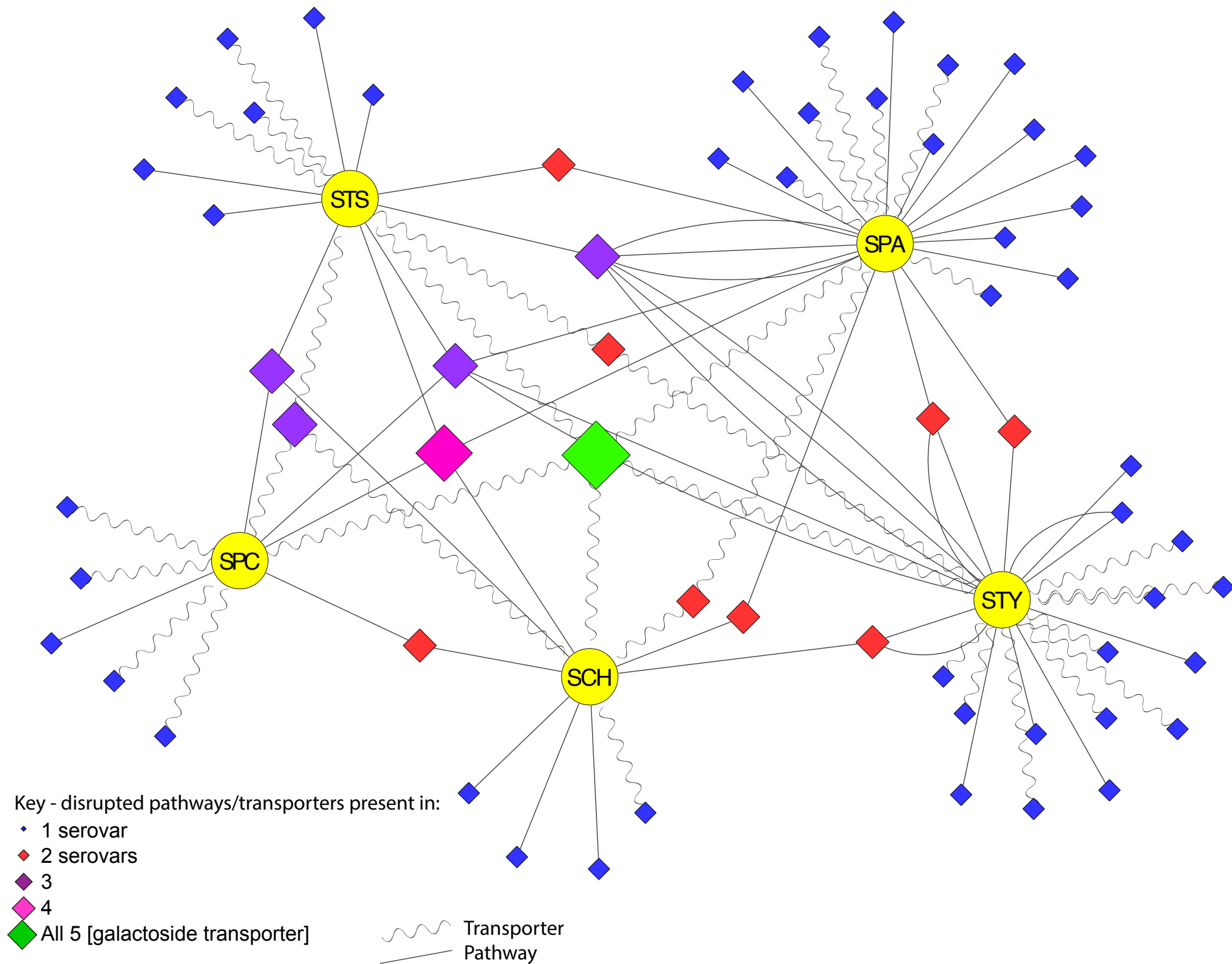

Supplement: Supplementary file 1 [file microorganisms-08-00657-s001.zip › Figure S2 lesions in pathways transporters.pdf]
